# Diagnostic challenges of renal medullary carcinoma and the role for cytologic assessment: Case report and literature review

**DOI:** 10.1002/jcla.24854

**Published:** 2023-02-26

**Authors:** Benjamin O. Severseike, Kristian T. Schafernak, Scott D. Willard, Luis F. Goncalves, Alok K. Kothari, Francis K. Eshun, Ross Mangum

**Affiliations:** ^1^ Phoenix Children's Hospital Residency Program Alliance (PCHRP) Phoenix Children's Hospital Phoenix Arizona USA; ^2^ Pathology and Laboratory Medicine Phoenix Children's Hospital Phoenix Arizona USA; ^3^ Department of Child Health University of Arizona College of Medicine Phoenix Arizona USA; ^4^ Creighton University School of Medicine Phoenix Arizona USA; ^5^ Mayo Clinic Alix School of Medicine Phoenix Arizona USA; ^6^ Interventional Radiology Phoenix Children's Hospital Phoenix Arizona USA; ^7^ Radiology Department Phoenix Children's Hospital Phoenix Arizona USA; ^8^ Center for Cancer and Blood Disorders Phoenix Children's Hospital Phoenix Arizona USA

**Keywords:** fine‐needle aspiration, pediatrics, renal medullary carcinoma, sickle cell trait, *SMARCB1* (*INI*)

## Abstract

**Background:**

Renal medullary carcinoma (RMC) is a diagnostically challenging, aggressive primary renal malignancy associated with abysmal survival. Delays in diagnosis contribute to most patients having diffusely metastatic disease at the time of initial presentation.

**Methods:**

We present the case of a 13‐year‐old African American male with sickle cell trait who presented with a renal mass and hematuria. Evaluation included imaging, fluid cultures, and cytologic assessment.

**Results:**

Patient was diagnosed with RMC based on cytologic assessment of sub‐centimeter fluid collections aspirated from the left kidney at the time of cortical biopsy for suspected renal mass. The additional fluid aspiration in conjunction with renal biopsy was an atypical but crucial step in early diagnosis.

**Conclusion:**

Cytomorphologic evaluation of fluid biospecimens is not currently part of the standard work‐up for patients with renal masses but, when available, can provide crucial information that reduces time to diagnosis. Prompt symptom recognition and treatment initiation may improve patient outcomes.

## 1 INTRODUCTION

Renal medullary carcinoma (RMC) is a rare primary renal malignancy typically affecting young males of African descent with sickle cell trait or other sickle hemoglobinopathies.^1^, ^2^, ^3^ Definitive diagnosis of RMC can be challenging due to its nonspecific radiographic features, lack of localizing symptoms, and overlapping features with other primary renal malignancies. Due to the inherently aggressive nature of RMC, most patients present with metastatic disease involving regional lymph nodes, the liver, lungs, bones, and/or adrenal glands and consequently face poor overall survival (OS).^4^, ^5^ High‐clinical suspicion is necessary for prompt diagnosis and treatment. Herein, we report the case of a 13‐year‐old African American male who presented with concern for acute pyelonephritis and was unexpectedly diagnosed with RMC based on cytology from aspirated fluid collections obtained in conjunction with a renal cortical biopsy.

## 2 CASE REPORT

**FIGURE 1:**
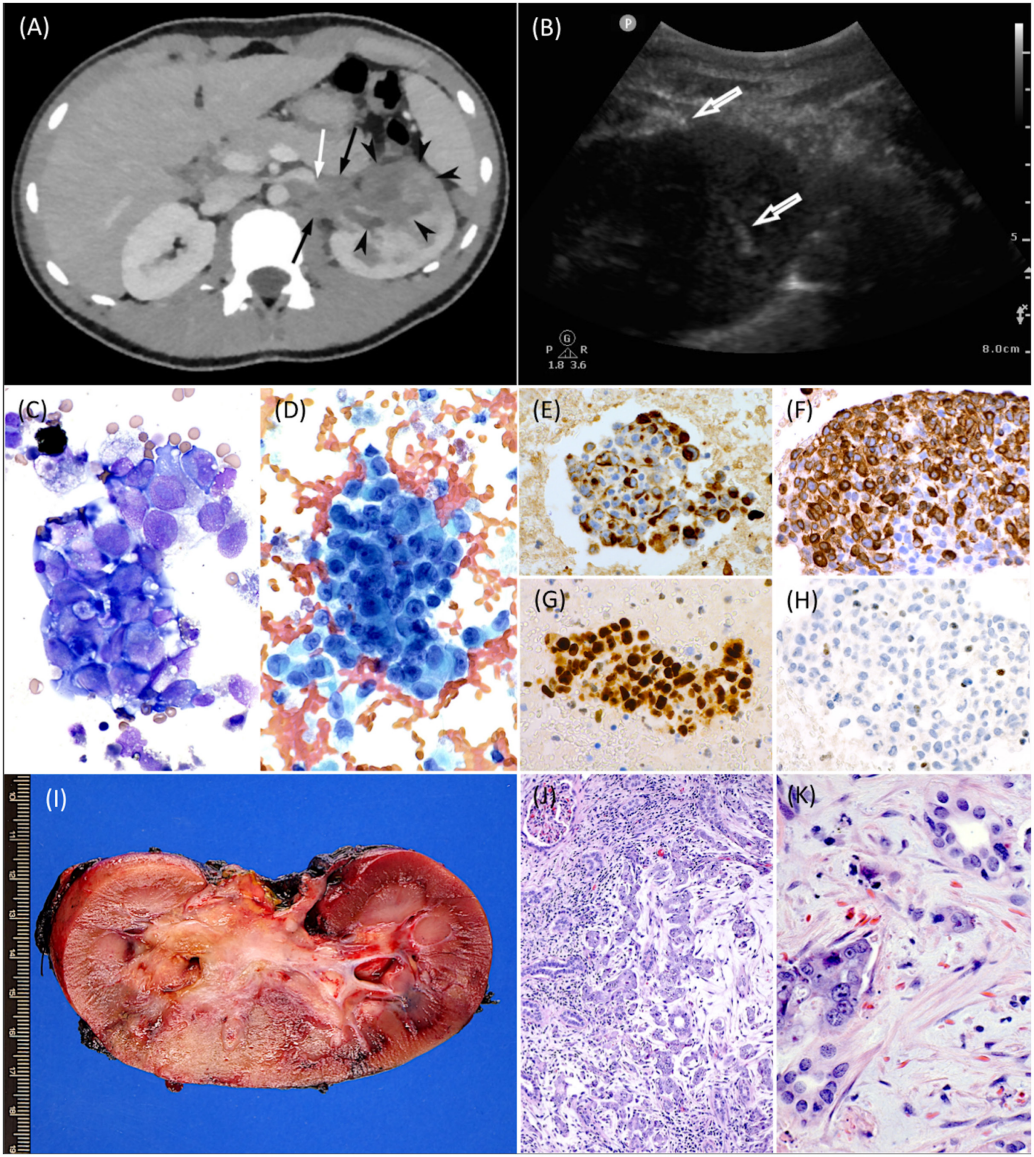
(A) Contrast‐enhanced CT with an hypoattenuating infiltrative mass in the interpolar region of the left kidney (black arrowheads) with extrarenal extension through the hilum (black arrows). Abrupt contrast cutoff with beaking in the renal vein was consistent with thrombosis (white arrow). At the time of this study, the swelling, hypoattenuation and wedge‐shaped perfusion defect in the kidney and adjacent adenopathy were interpreted as suggestive of an atypical infection such as tuberculosis or coccidioidomycosis, with two lower pole fluid collections possibly representing abscesses. (B) Grayscale ultrasound image of the left renal pole with aspiration needle in place (arrows); the needle tip is in one of the two complex subcentimeter hypoechoic fluid collections sampled at the same time as core biopsies for cultures in our microbiology laboratory and histology at a reference laboratory specializing in medical renal disease. Cytospin preparations of the fluid showed clusters of tumor cells with variable amounts of vacuolated cytoplasm (C, Wright‐Giemsa, 400×) and irregular nuclei containing prominent nucleoli (D, Papanicolaou, 400×). Immunostains performed on the cell block material revealed positivity of many tumor cells for keratin AE1/AE3 (E, 400×; note paranuclear globular staining in a subset of cells with rhabdoid features) and vimentin (F, 400×), a very high Ki67/MIB1 proliferation index (G, 400×), and loss of nuclear INI1 expression in most cells (H, 400×). The left nephrectomy specimen (I) demonstrated an 8.4 cm ill‐defined tumor present in the mid to lower pole, centered on the medulla and extending into the renal sinus, with a positive renal vein margin. Histologic sections (J, H&E, 100×) revealed a variety of architectural patterns including a reticular or lace‐like pattern, islands/nests, ribbons/trabeculae, a cribriform adenoid cystic carcinoma‐like pattern with necrosis associated with neutrophils, and a microcystic pattern. Sickled erythrocytes were seen in the tissue as well (K, H&E, 400×).

A 13‐year‐old African American male with sickle cell trait presented with hematuria and left flank pain following a failed course of antibiotics for a presumed urinary tract infection. Renal ultrasound demonstrated bilateral heterogeneously hyperechogenic kidneys with loss of corticomedullary differentiation suggestive of acute pyelonephritis. Subsequent computed tomography (CT) revealed a markedly abnormal appearance of the left kidney (Figure 1A). Given the presence of two fluid collections in the lower pole consistent with abscess formation, an atypical infection was favored.

A renal cortical biopsy demonstrated nonspecific, protracted acute tubular injury and interstitial fibrosis without malignant cells. Fortunately, aspiration of the two lower pole fluid collections was also performed (Figure 1B) with cytology demonstrating atypical cells with vacuolated cytoplasm and prominent nucleoli as well as positivity for keratin AE1/AE3, vimentin, a high Ki67/MIB1 proliferation index, and loss of nuclear INI1 expression (Figure 1C–H). In the setting of a young patient with sickle cell trait, these findings suggested a diagnosis of RMC. There was an excellent cytologic‐histologic correlation with the radical nephrectomy specimen (Figure 1I–K). Metastatic disease was confirmed by the presence of multiple bilateral pulmonary nodules on chest CT.

The patient received systemic chemotherapy with carboplatin, gemcitabine, and paclitaxel therapy for three cycles. Due to the development of acute kidney injury, he was switched to tazemetostat. After two cycles, positron emission tomography (PET) showed a significant decrease in the size and number of the pulmonary nodules. However, follow‐up CT scan 6 months later revealed progression of his pulmonary metastases. Subsequently, he was started on oral erlotinib and intravenous bevacizumab, with decreased size and reduced fluorodeoxyglucose avidity of the pulmonary nodules on his most recent scans 20 months from diagnosis.

## 3 DISCUSSION

Patients with RMC most often present in the second to third decade of life.^6^, ^7^ In the pediatric age group, the median age at diagnosis is 13 years.^8^ The large majority of patients with RMC have sickle cell trait or other related hemoglobinopathies. The pathophysiologic mechanism driving RMC tumorigenesis remains elusive. A leading hypothesis posits that a chronically hypoxic environment in the renal pelvis exacerbates the sickling of cells, triggering local inflammation, necrosis, cellular damage, and eventually the development of RMC from the calyceal epithelium.^4^, ^9^ The hallmark molecular alteration seen in RMC is inactivation of the *SMARCB1* (*INI*) tumor suppressor gene on chromosome 22 due to chromosomal translocations or deletions.^6^, ^9^, ^10^, ^11^, ^12^

The typical presenting symptoms of RMC include hematuria, flank pain, and/or an abdominal mass.^4^ Given the aggressive nature of the disease, systemic symptoms such as weight loss and fatigue are common findings.^24^ Most patients present with evidence of metastatic disease at diagnosis. In a pooled analysis of 166 patients, 71% had at least localized metastasis.^24^ The regional lymph nodes, liver, lungs, bone, and adrenal glands comprise the most common sites of metastasis. Some patients may have a suspected renal abscess or urinary tract infection and may present without a clinically recognizable mass.

Due to this variable presentation, full body screening remains imperative to staging RMC. The diagnostic work‐up typically consists of radiographic imaging with CT of the chest, abdomen, and pelvis often demonstrating a unilateral renal mass with distant metastatic spread. However, the lack of clearly defined radiographic features and the absence of a standardized imaging approach complicates the diagnosis of RMC.^8^, ^13^ Lesions often appear as hypovascular, infiltrative, poorly defined solid masses occupying the renal medulla and lying adjacent to the pelvicalyceal system.^14^ Given the rare nature of RMC, radiologists may not consider it in their standard differential for a renal mass. Thus, a high degree of clinical suspicion must be maintained, particularly in a patient with a known or suspected sickle‐related hemoglobinopathy.

RMC can arise in the medulla or the cortex and assume a variety of architectural growth patterns, such as the reticular or lace‐like pattern, island/nests, ribbons/trabeculae, cribriform adenoid cystic carcinoma‐like pattern with necrosis (associated with neutrophils), or the microcystic pattern we observed in our case. The tumor cells (a subset of which had rhabdoid features) were also partially set in a myxoid stroma and focally associated with a lymphocytic infiltrate. These are all common features. Consistently, the tumor cells were positive for PAX8, broad‐spectrum keratins, epithelial membrane antigen, and vimentin. They were also negative for INI1, though they are certainly not the only tumor (nor the only renal tumor) to show INI1 loss.^15^

**FIGURE 2:**
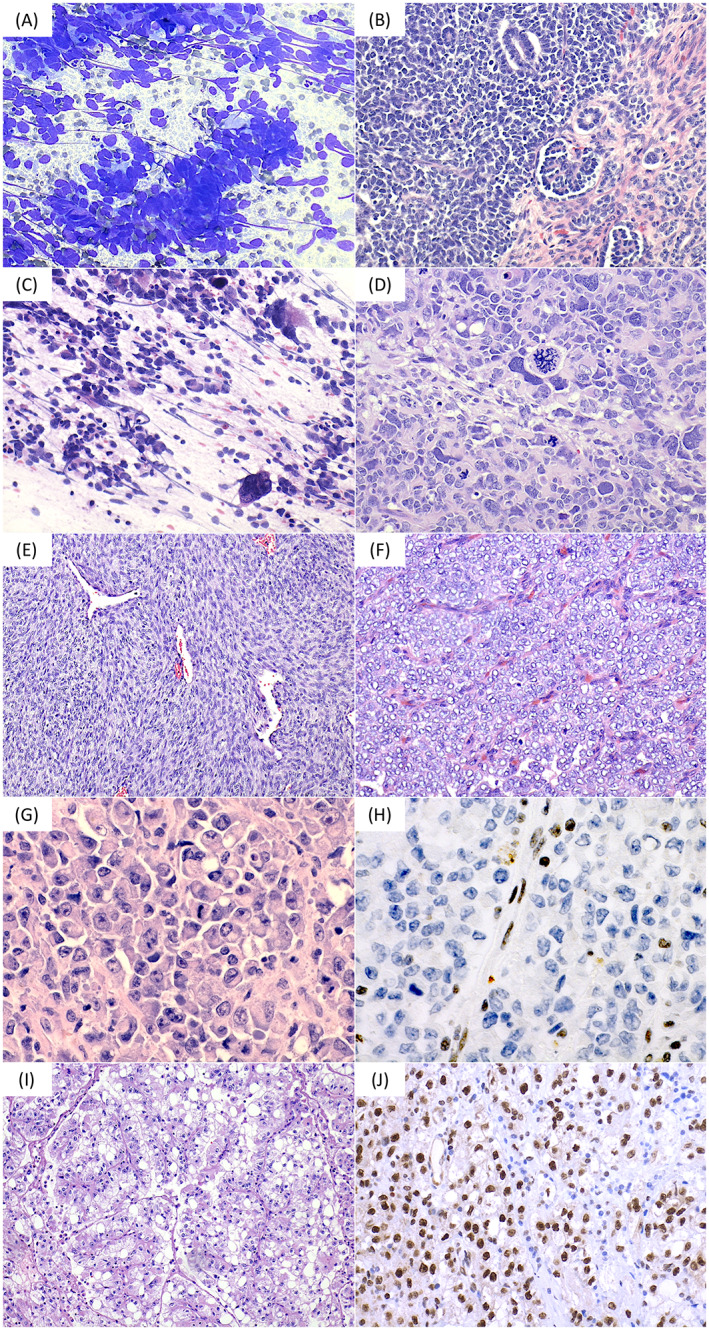
Most common pediatric renal tumors. (A) Fine‐needle aspirate of Wilms tumor without anaplasia (Diff‐Quik, 200×). The majority of cells are blastemal, with scant cytoplasm and overlapping, round to angulated nuclei. (B) Histology of Wilms tumor without anaplasia (H&E, 200×) demonstrating triphasic pattern comprising blastemal, epithelial (tubular and glomerular elements), and stromal components. Mean age is 3–4 years, though rare cases present during adulthood. (C) Touch imprint of Wilms tumor with diffuse anaplasia showing pleomorphic, hyperchromatic, enlarged nuclei (H&E, 200×). (D) Histology of Wilms tumor with anaplasia (H&E, 200×). Note large hyperchromatic nuclei three times the length of nonanaplastic nuclei as well as multipolar mitotic figures. (E) Congenital mesoblastic nephroma (CMN; H&E, 100×) composed of interlacing fascicles of spindle cells, with an hemangiopericytoma‐like vascular pattern. Aspirates of the classic type of CMN are frequently non‐diagnostic, while cellular CMN yields greater cellularity, with slightly pleomorphic, ovoid to polygonal cells containing coarse chromatin. Most patients are less than 2 years old. (F) Clear cell sarcoma of kidney (H&E, 200×) with nests and cords of cells with nuclei containing fine chromatin and lacking prominent nuclei, separated by regularly spaced septa with arborizing “chicken‐wire” capillaries. Smears show clustered or dispersed tumor cells with indented/grooved nuclei, sometimes set in a myxoid or mucoid background. Mean age is 3 years. (G) Rhabdoid tumor is highly aggressive, tending to arise in the medulla and present before age 4 ears (H&E, 400×). Tumor cells contain vesicular chromatin, prominent nucleoli and hyaline pink cytoplasmic inclusions. Rhabdoid features can actually be seen in many pediatric tumors. Given the location of the tumor and loss of nuclear INI1 (BAF47) expression (H; INI1, 400×), rhabdoid tumor is the closest differential diagnosis to RMC, though the latter occurs in older patients. (I; H&E, 100×) Renal cell carcinoma (RCC)—this particular case is an MiT family translocation RCC with t(X;1)(p11.2;q21.2) on chromosome analysis, fusing *PRCC* and *TFE3* and resulting in strong nuclear immunoreactivity for the transcription factor, TFE3 (J; 200×). Usual cases have papillary architecture, epithelioid clear cells, and abundant psammomatous calcifications.

The differential diagnosis of RMC can be organized based on age,^16^ location,^9^ cytologic features,^17^ and loss of nuclear SMARCB1/INI1 expression^15^, ^18^, ^19^, ^20^ (Figure 2, Table 1). The new World Health Organization Classification of Urinary and Male Genital Tumors now classifies RMC as “SMARCB1‐deficient renal medullary carcinoma”.^16^ Patients with RMC typically have sickle cell trait, though a smaller number of patients have had hemoglobin SC or homozygous SS disease. In the absence of such a history, one should recommend hemoglobin electrophoresis/high‐performance liquid chromatography. Yet there are rare cases defying classification that are morphologically identical to RMC but occur in the absence of a demonstrable hemoglobinopathy. Such cases were previously called “unclassified renal cell carcinoma with medullary phenotype” and currently can be regarded as a subtype of “SMARCB1‐deficient RCC with medullary‐like features or phenotype”. It should also be pointed out that SMARCB1 protein loss can occur as a secondary event, as in rare cases of collecting duct carcinoma, fumarate hydratase‐deficient renal cell carcinoma, or unclassifiable renal cell carcinoma, that should be classified by their primary tumor type and qualified “with secondary SMARCB1 loss”.^16^ The significant morphologic and immunophenotypic overlap between renally based tumors can lead to diagnostic delays, especially in the setting of insufficient tumor tissue to complete comprehensive testing. In these scenarios, consideration of key patient demographic factors—age, race, and sickle cell trait status—is particularly important. Testing biospecimens such as effusions or renal fluid collections can significantly and expeditiously increase diagnostic accuracy and confidence.

**TABLE 1:** Differential diagnosis of SMARCB1‐deficient renal medullary carcinoma based on age, location, cytologic features, and loss of nuclear SMARCB1/INI1 expression.

Most common pediatric renal tumors in order of descending frequency^15^ (illustrated in Figure 2)	
75%–80%	Wilms tumor (nonanaplastic) (mean age, 3–4 years; rare cases during adulthood)
5%–8%	Wilms tumor (anaplastic)
4%	Congenital mesoblastic nephroma (most patients less than 2 years old)
3%–4%	Clear cell sarcoma of the kidney (mean age, 3 years)
3%–4%	Renal cell carcinoma
2%	Rhabdoid tumor (unusual above age 3 years)
1%–2%	Miscellaneous (includes neuroblastoma, Ewing sarcoma, synovial sarcoma, angiomyolipoma, lymphoma, other)
Medullary based renal masses^9^	
Previous “unclassified renal cell carcinoma with medullary phenotype” morphologically identical to renal medullary carcinoma but without any hemoglobinopathy (now regarded as a subtype of SMARCB1‐deficient renal cell carcinoma with medullary‐like features or phenotype)	
Collecting duct carcinoma	
Upper tract urothelial carcinoma	
Fumarate hydratase‐deficient renal cell carcinoma	
Metastatic carcinoma/melanoma	
*ALK*‐rearranged renal cell carcinoma	
High‐grade papillary renal cell carcinoma	
Lymphomas/lymphoproliferative disorders	
Xanthogranulomatous pyelonephritis	
Cytologic differential diagnosis^20^	
*Renal medullary carcinoma*: loosely cohesive or two‐dimensional clusters and individual cells (no true papillary structures) with an epithelial appearance, high nuclear: cytoplasmic ratio, often eccentrically placed nuclei, marked nuclear pleomorphism, irregular nuclear membranes, finely granular to vesicular chromatin, one to several prominent nucleoli, and dense, frequently vacuolated cytoplasm with sharp cytoplasmic borders	
*Collecting duct carcinoma*: two‐ or three‐dimensional groups of cells with papillary configuration, moderate nuclear pleomorphism, irregular nuclear membranes, coarse chromatin, prominent nuclei, and vacuolated cytoplasm	
*Urothelial carcinoma*: clusters and individual cells, large irregular eccentrically placed hyperchromatic nuclei, coarse chromatin, and dense cytoplasm; “cercariform” cells have long cytoplasmic tails	
*Renal cell carcinoma*: large clusters, low nuclear: cytoplasmic ratio, uniform nuclei, fine chromatin, inconspicuous nucleoli, and abundant clear or granular cytoplasm	
*Metastatic adenocarcinoma* (particularly from lung, in pleural effusions): three‐dimensional tight clusters and acinar structures, irregular nuclear membranes, coarse chromatin, prominent nucleoli, vesicular and lacy cytoplasm, sometimes containing mucin, indistinct cell borders; usually occurs in older patients	
Other tumors that can be SMARCB1/INI1‐deficient^15^, ^21^, ^22^, ^23^, ^24^	
Rhabdoid tumor of kidney (usually presents before age 4 years; diffuse infiltrative growth with diverse pattern of immunoreactivity for keratins and vimentin as well as smooth muscle actin, synaptophysin, glial fibrillary acidic protein, and in over 50%, CD99)	
Collecting duct carcinoma with secondary SMARCB1 loss (tubular/tubulopapillary architecture most common and typically positive for high‐ and low‐molecular‐weight keratins and PAX8; some cases previously diagnosed as collecting duct carcinoma have been reclassified as fumarate hydratase‐deficient renal cell carcinoma)	
Fumarate hydratase‐deficient renal cell carcinoma with secondary SMARCB1 loss (can show various architectural patterns, most frequently papillary, followed by solid, tubulocystic, cribriform/sieve‐like, and cystic; look for loss of fumarate hydratase and/or expression of 2‐succinocysteine by immunohistochemistry)	
Unclassified renal cell carcinoma with secondary SMARCB1 loss	
Atypical teratoid/rhabdoid tumor	
Epithelioid sarcoma, both classic and proximal‐type	
SWI‐deficient sinonasal carcinoma (SMARCB1‐deficient carcinoma/adenocarcinoma)	
Poorly differentiated chordoma	
Myoepithelial carcinoma of soft tissue	
Epithelioid schwannoma and malignant peripheral nerve sheath tumor	
Extraskeletal myxoid chondrosarcoma	
Rhabdoid carcinoma of the gastrointestinal tract and pancreas	
Undifferentiated and dedifferentiated carcinoma of the ovary and uterine corpus	
Desmoplastic myxoid tumor of the pineal region, *SMARCB1*‐mutant	
Cribriform neuroepithelial tumor (CRINET)	

Due to the tendency for the advanced stage of disease at presentation and the inherent aggressive behavior of most cases of RMC, survival outcomes remain poor despite aggressive multimodal treatment with radical nephrectomy, chemotherapy, and occasionally radiotherapy. The overall survival for RMC is abysmal with one study citing OS of 4.7 months for patients with metastatic disease and 17.8 months for patients with localized disease.^5^ Ezekian et al. analyzed survival outcomes for a cohort of 159 RMC patients and found the median survival of all patients to be 7.7 months with a 5‐year OS of 9%. While no patients with metastatic disease were alive at 5 years, seven patients without metastatic disease achieved this benchmark and were identified as long‐term survivors. All long‐term survivors presented with localized disease and underwent successful radical nephrectomy with negative margins.^5^ This emphasizes the critical importance of timely symptom recognition to facilitate the earliest possible intervention. Prompt treatment initiation with radical nephrectomy and aggressive systemic chemotherapy prior to disease dissemination may portend higher likelihood of survival.

This case highlights a key diagnostic challenge associated with tissue biopsy in cases of RMC. The majority of kidney disease processes manifest in the renal cortex. Medullary biopsies carry a higher risk for complications such as hemorrhage, worsening hematuria, arterio‐venous fistula, and urinoma.^19^ The cortical biopsies sent from our patient to a reference laboratory specializing in medical renal disease were determined to be negative for malignancy. Therefore, it was aspiration cytology of the lower pole fluid collections, taken into consideration with critical pieces of the medical history—age, sex, and mostly importantly, sickle cell trait—that led to the diagnosis. Previous cases have shown that RMC can be diagnosed by cytologic examination of urine, renal pelvic washings, fine‐needle aspiration of the kidney, and serous cavity effusions.^17^, ^22^, ^23^, ^24^ Miller et al. analyzed 12 patients with RMC presenting with serous cavity effusions. For all patients, cytologic fluid assessment played a key role in narrowing the differential and ultimately establishing a final diagnosis of RMC either by prompting additional radiologic studies that identified the primary renal tumor or by confirming loss of INI1 expression in tumor cells.^24^

## 4 CONCLUSION

RMC is a diagnostically challenging tumor requiring a high degree of suspicion and astute clinical recognition of the association of sickle cell trait with RMC. As highlighted by this case study, procurement and cytologic examination of any effusions or other fluid specimens may potentially expedite the diagnostic process. Proactively pursuing such diagnostic modalities, as opposed to awaiting definitive histopathologic characterization, should be aggressively pursued.

## AUTHOR CONTRIBUTIONS

Severseike – Manuscript conceptualization, manuscript preparation of entire text, manuscript revisions. Schafernak – Manuscript conceptualization, preparation of cytologic images and discussion in text, manuscript revisions. Willard – Contributed to manuscript preparation for sections discussing interventional radiology procedures, manuscript revisions. Goncalves – Contributed all radiologic images and discussion of radiologic findings, manuscript revisions. Kothari – Content expertise, manuscript revisions. Eshun – Context expertise, manuscript revisions. Mangum – Manuscript conceptualization, manuscript preparation of entire text, manuscript revisions.

## CONFLICT OF INTEREST STATEMENT

No authors have conflicts of interest to declare.

## Data Availability

The data that support the findings of this study are available from the corresponding author upon reasonable request.
